# Activity of methylgerambullin from *Glycosmis* species (Rutaceae) against *Entamoeba histolytica* and *Giardia duodenalis in vitro*

**DOI:** 10.1016/j.ijpddr.2019.08.001

**Published:** 2019-08-10

**Authors:** Mirjana Drinić, Adriane Raninger, Andrea Zraunig, Florian Astelbauer, David Leitsch, Andreas Obwaller, Julia Walochnik, Harald Greger, Michael Duchene

**Affiliations:** a Institute of Specific Prophylaxis and Tropical Medicine, Center for Pathophysiology, Infectiology and Immunology, Medical University of Vienna, A-1090, Vienna, Austria; b Chemodiversity Research Group, Faculty of Life Sciences, University of Vienna, A-1030, Vienna, Austria; c Orphanidis Pharma Research GmbH, A-1160, Vienna, Austria; d CDMS Clinical Data Management and Statistics GmbH, A-1020, Vienna, Austria

**Keywords:** *Entamoeba histolytica*, *Giardia duodenalis*, *Glycosmis* spp., Methylgerambullin, Sulphur-containing amide, Aglafoline

## Abstract

*Entamoeba histolytica* and *Giardia duodenalis* are widespread intestinal protozoan parasites which both spread via cysts that have to be ingested to infect a new host. Their environment, the small intestine for *G. duodenalis* and the colon for *E. histolytica*, contains only very limited amounts of oxygen, so both parasites generate energy by fermentation and substrate level phosphorylation rather than by oxidative phosphorylation. They both contain reducing agents able to reduce and activate nitroimidazole drugs such as metronidazole which is the gold standard drug to treat *Entamoeba* or *Giardia* infections. Although metronidazole works well in the majority of cases, it has a number of drawbacks. In animal models, the drug has carcinogenic activity, and concerns about a possible teratogenic activity remain. In addition, the treatment of *G. duodenalis* infections is hampered by emerging metronidazole resistance. Plant-derived drugs play a dominant role in human medicine, therefore we tested the activity of 14 isolated plant compounds belonging to seven different classes *in vitro* against both parasites. The tests were performed in a new setting in microtiter plates under anaerobic conditions. The compound with the highest activity was methylgerambullin, a sulphur-containing amide found in *Glycosmis* species of the family Rutaceae with an EC_50_ of 14.5 μM (6.08 μg/ml) after 24 h treatment for *E. histolytica* and 14.6 μM (6.14 μg/ml) for *G. duodenalis*. The compound was successfully synthesised in the laboratory which opens the door for the generation of new derivatives with higher activity.

## Highlights

• Fourteen plant compounds of seven classes were isolated.
• The activity against *Entamoeba histolytica* and *Giardia duodenalis* was tested.
• Methylgerambullin had the highest activity against both parasites.
• Methylgerambullin is a sulphur-containing amide from *Glycosmis* spp. (*Citrus* plants).
• High cysteine concentrations interfered with methylgerambullin activity.

## Graphical abstract

**Figure:**
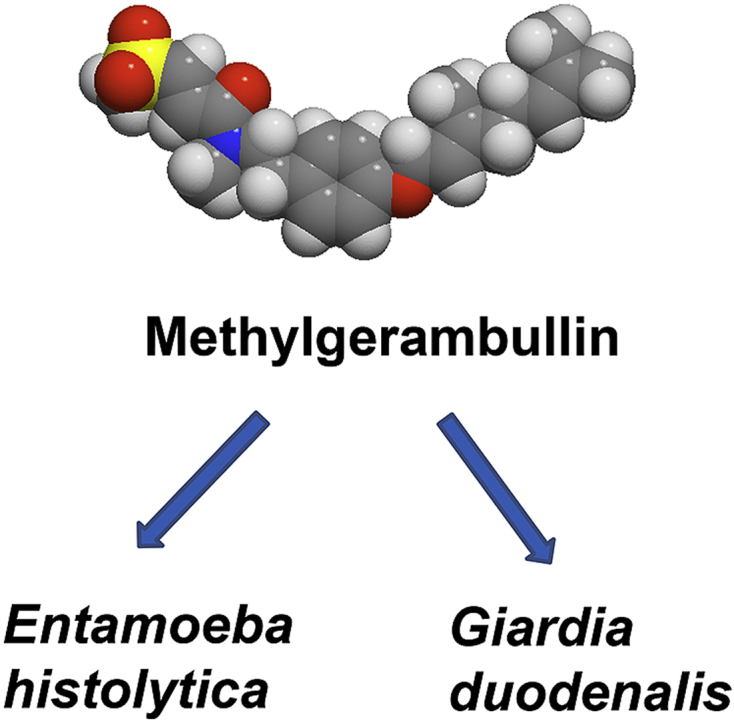

## 1 Introduction

*Entamoeba histolytica* and *Giardia duodenalis* are human protozoan parasites with a simple life cycle lacking intermediate hosts. Infectious cysts are passed in the stool of patients and have to reach uninfected individuals either via smear infection or via food or water. After passing through the stomach, they excyst as trophozoites. Whereas *G. duodenalis* trophozoites colonize the small intestine, *E. histolytica* trophozoites reside in the colon.

*G. duodenalis* infections can remain without symptoms, but can also lead to diarrhoea with greasy or foul-smelling stools, accompanied by abdominal pain, flatulence, bloating, nausea, and sometimes weight loss (Minetti et al., 2016). As the parasite interferes with the absorption of nutrients in the small intestine, it is not surprising that cases of giardiasis were associated with underweight and severe malnutrition in children in a study from Rwanda (Ignatius et al., 2012). An estimated 184 million of symptomatic cases per year occur worldwide (Havelaar et al., 2015), with a higher frequency in poor regions with a lack of clean drinking water. Deaths caused by *G. duodenalis* infections are extremely rare (Gargano et al., 2017).

In the colon, *E. histolytica* trophozoites are able to phagocytose bacteria and take up remaining nutrients from the host, thus the infection can remain asymptomatic, but they can also attach to the mucus and enterocytes and penetrate the protective layers into the intestinal wall. This can result in amoebic dysentery with abdominal pain, tenesms and diarrhoea, sometimes with blood-covered stools. Moreover, the amoebae can invade the mesenterial vessels to be carried to the liver where they can establish large abscesses (Stanley, 2003). The Global Burden of Disease 2010 Study of the University of Washington estimated 55,500 deaths and 2.2 million years of life lost from premature death or disability (DALYs) caused by amoebiasis (Turkeltaub et al., 2015). So amoebiasis remains a serious neglected infectious disease.

Both *E. histolytica* and *G. duodenalis* infections are treated with metronidazole, as the gold standard drug. To become active, the drug must be reduced at its nitro group (Müller, 1983). This reduction typically occurs in microaerophilic or anaerobic microorganisms. *E. histolytica* and *G. duodenalis* possess a key enzyme, pyruvate:ferredoxin oxidoreductase (PFOR) catalysing the oxidation of pyruvate to acetyl-CoA and CO_2_ with the concomitant generation of reduced ferredoxin, which is able to activate metronidazole (Moreno et al., 1984; Upcroft and Upcroft, 2001). An alternative mechanism to reduce and activate metronidazole is by thioredoxin reductase with the cofactor NADPH (Leitsch et al., 2007). In addition, a nitroreductase GINR1 with the ability to reduce metronidazole has been characterised in *G. duodenalis* (Nillius et al., 2011).

Considering that metronidazole has been used for more than 50 years in *E. histolytica*, the low level of resistance is surprising. Treatment failures have been reported (Hanna et al., 2000), and in the laboratory the amoebae could be adapted to metronidazole concentrations between 10 μM (Upcroft and Upcroft, 2001) and 40 μM (Wassmann et al., 1999), but to our knowledge, no resistant strain could be isolated from any patient. In *G. duodenalis*, metronidazole resistance is much more of a problem (Upcroft and Upcroft, 2001; Leitsch et al., 2012).

Metronidazole treatment is associated with some common adverse effects such as metallic taste, headache, nausea, and negative interaction with alcohol, and rarely, with central or peripheral neurotoxicity, pancreatitis or neutropenia (Gardner and Hill, 2001). The biggest concern, however, is that the International Agency for Research on Cancer (IARC) has listed metronidazole as animal carcinogenic and possible human carcinogenic (IARC, 1987). DNA damage has been observed in individuals treated with metronidazole, however, the exact mechanism how this occurs remains unknown, and no long-term studies have been carried out to resolve the question if metronidazole is carcinogenic for humans (Bendesky et al., 2002). Taken together, emerging resistance in the case of *G. duodenalis* and remaining concerns over its possible carcinogenic activity justify to continue the search for alternatives to metronidazole.

Throughout recorded human history, medicines derived from plants have been used to treat various diseases (Cragg and Newman, 2013), in particular infections with parasites (Wink, 2012). Malaria treatment by quinine, its derivatives chloroquine and mefloquine, and the current drug artemisine (Tu, 2016) and its derivatives has literally saved many millions of lives. These drugs, like all anti-infective agents, suffer from problems of resistance, even the best of them, artemisinin (Noedl et al., 2008), so the search has to continue. New compounds from plants often have complex structures, are extracted in small amounts and the ownership of intellectual property may raise disputes. In the last two decades of the previous century, high-throughput synthesis of compounds addressing very specific targets was hoped to quickly generate better drug candidates. With some disappointments of the pure chemical approach, and with a realistic view on drug development from plants, this field recovered in the new century (Balunas and Kinghorn, 2005). In a large review, Newman and Cragg (2016) analysed the 1562 new drugs approved in the period between 1981 and 2014. These included only 27% of chemically synthesised drugs. The vast majority is a complex list of pure or mixed natural products, mostly derived from plants, chemically modified natural products, synthetic drugs with a natural pharmacophore, mimics of natural products, as well as vaccines.

Previously we tested the activity of a small series of plant-derived compounds comprising several classes against several important protozoan parasites. The maturation of *Plasmodium falciparum* schizonts was inhibited by sub-micromolar concentrations of the flavaglines rocaglamide and aglafoline (Astelbauer et al., 2012). Two further studies, included, in addition, sulphur-containing amides from *Glycosmis* spp. (Rutaceae). These compounds were highly active in low micromolar concentrations against *Trypanosoma cruzi* epimastigotes (Astelbauer et al., 2010) and *Leishmania infantum* promastigotes (Astelbauer et al., 2011). In the present work we tested the activity of a similar set of 14 compounds against *E. histolytica* and *G. duodenalis* and found the sulphur-containing amide methylgerambullin to display the highest activity. This compound is easily accessible to chemical synthesis opening the door to study its mechanism of action and to generate improved derivatives.

## 2 Materials and Methods

### 2.1 Parasites and culture

The *Entamoeba histolytica* trophozoites (strain HM-1:IMSS, ATCC 30459) used in this study were axenically cultivated in TYI-S-33 medium (Diamond et al., 1978), containing 10% (v/v) complement-inactivated bovine serum, 1% (v/v) penicillin/streptomycin solution (10,000 units penicillin and 10 mg streptomycin per ml, Sigma-Aldrich) and 3% (v/v) of complete vitamin mixture (Diamond Vitamin Tween 80 Solution, SAFC Biosciences, KA, USA). Axenical cultivation of *Giardia intestinalis* WB clone 6 (ATCC 50803) cells was performed in Keister's modified TYI-S-33 medium (Keister, 1983), supplemented with 1% (v/v) penicillin/streptomycin solution and 10% (v/v) complement-inactivated bovine serum. Both media are rich in cysteine, 1 mg/ml for *E. histolytica* and 2 mg/ml for *G. duodenalis*. *Entamoeba* trophozoites were subcultured twice and *Giardia* trophozoites three times per week.

### 2.2 Compounds

**Fig. 1:**
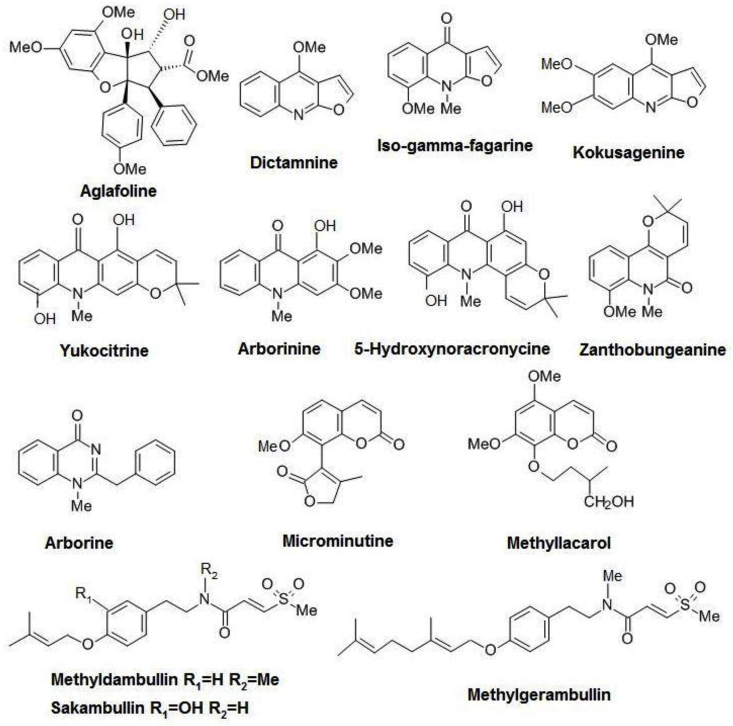
The 14 compounds tested against *E. histolytica* and *G. duodenalis* in this study.

The fourteen tested compounds (Fig. 1, Table 1) belong to seven different chemical classes. Aglafoline is a flavagline from the family Meliaceae (mahogany family). The furoquinolines dictamnine, iso-gamma-fagarine and kokusagenine, the acridones yukocitrine, arborinine and 5-hydroxynoracronycine, the quinolinone zanthobungeanine and the quinazoline arborine are alkaloids found in members of the family Rutaceae (*Citrus* plants). Methyllacarol found in Asteraceae and microminutine from Rutaceae are coumarins, finally the sulphur-containing amides methyldambullin, sakambullin and methylgerambullin are again found in Rutaceae.

**Table 1:** List of compounds tested in this study, compound class, origin from plant family and species, plant organs, references.

Compound	Compound class	Plant family	Plant species	Plant organ	References for isolation
Aglafoline	Flavagline	Meliaceae	*Aglaia odorata*	Stembark	Brem. (2002)
					Greger et al. (2001)
Dictamnine	Furoquinoline	Rutaceae	*G. trichanthera*	Rootbark	Vajrodaya et al. (1998)
Iso-gamma-fagarine	Furoquinoline	Rutaceae	*G. sapindoides*	Leaves	Vajrodaya (1998)
Kokusagenine	Furoquinoline	Rutaceae	*G. sapindoides*	Rootbark	Vajrodaya (1998)
Yukocitrine	Acridone	Rutaceae	*G. trichanthera*	Stembark	Vajrodaya et al. (1998)
Arborinine	Acridone	Rutaceae	*G. sapindoides*	Leaves	Vajrodaya (1998)
5-Hydroxy-noracronycine	Acridone	Rutaceae	*G. trichanthera*	Stembark	Vajrodaya et al. (1998)
Zanthobungeanine	Quinolinone	Rutaceae	*Zanthoxylum simulans*	Rootbark	Brader et al. (1993)
Arborine	Quinazoline	Rutaceae	*G. pentaphylla*	Leaves	Vajrodaya (1998)
Microminutine	Coumarin	Rutaceae	*Micromelum* cf*.minutum*	Leaves	Grassi (1998)
Methyllacarol	Coumarin	Asteraceae	*Artemisia laciniata*	Leaves	Szabo et al. (1985)
Methyldambullin	S-amide^a^	Rutaceae	*G. angustifolia*	Leaves	Greger et al. (1994)
Sakambullin	S-amide^a^	Rutaceae	*G. chlorosperma*	Leaves	Hofer et al. (2000)
Methylgerambullin	S-amide^a^	Rutaceae	*G. trichanthera*	Leaves	Vajrodaya et al. (1998)

a S-amide = sulphur-containing amide.

The compounds were extracted from various plant organs (Table 1) as described before (Greger et al., 1994, 1996; Hofer et al., 2000). Briefly, the methanolic extract was concentrated and the aqueous residue extracted with CHCl_3_. The CHCl_3_ fractions were roughly separated by column chromatography (CC), and further separation was achieved by preparative medium pressure chromatography (MPLC). In some cases, preparative thin layer chromatography (TLC) was used for the final purification. The identity of the compounds was confirmed by comparison with authentic samples by high performance liquid chromatography (HPLC) and nuclear magnetic resonance (NMR) analysis. For stock solutions, the compounds were dissolved in dimethylsulphoxide DMSO (Sigma-Aldrich) at a concentration of 10 μg/ml.

### 2.3 Susceptibility assays

The assays were carried out in 96-well microplates. For *E. histolytica* assays, parasite viability was very poor, when the plates were only covered with parafilm and lid, or set in a candle jar as used in malaria research (Jensen and Trager, 1977) (results not shown). In contrast, cells remained viable and proliferated well in a rectangular air-tight plastic box with air-tight clamps (Komax, Korea) in which anaerobic conditions were ensured by Anaerocult A pouches (Merck Darmstadt, Germany). A box with the dimensions 19 cm length, 13 cm width and 4.5 cm height was suitable for up to two plates. Anaerobic conditions were tested with Anaerotest strips (Merck). The same system was also found suitable for *G. duodenalis* assays.

For the assays, the parasites were seeded at a concentration of 40,000 cells ml^−1^ in a volume of 300 μl. After incubation of 24 h or 48 h, to 20 μl of cells, released by vigorous pipetting, an equal volume of a 0.4% solution of Trypan blue (Sigma-Aldrich) was added and the number of the dead and living cells was counted in a Bürker-Türk haemocytometer.

Initially, the activity of the compounds was compared by quick tests with final drug concentrations of 2.5 μg/ml and 10 μg/ml. The tests were performed twice in triplicates and the percentage of growth inhibition GI was determined for each sample after incubation at 37 °C for 24 h or 48 h. GI [%] (percent growth inhibition) was calculated by the formula GI = [(Gc - Gp)/ Gc] x 100, where Gc equals the mean number of living cells per ml in control (no drug added), and Gp equals the mean number of living cells per ml at the different drug concentrations. Standard deviations σ_n_ were calculated.

When it turned out that methylgerambullin was by far the most active compound against *E. histolytica* and *G. duodenalis*, the compound was tested in the same manner, under the same conditions, only in a wider range of concentrations: 1 μg/ml, 2.5 μg/ml, 5 μg/ml, 7.5 μg/ml, 10 μg/ml and 20 μg/ml respectively. The EC_50_ (half maximal effective concentration) value of methylgerambullin was calculated *via* log-probit analysis (SPSS 16.0, IBM, Chicago, IL). All experiments were carried out three times with results counted in triplicates. The geometric means *G* [μM] of the EC_50_ values as well as the geometric standard deviations *σ*_*g*_ (Limpert et al., 2001) were calculated according to https://en.wikipedia.org/wiki/Geometric_standard_deviation. Metronidazole as positive reference compound was tested in the same way in two independent experiments and the EC_50_ values were calculated as well.

Finally, as aglafoline had also shown relevant activity against *G. duodenalis*, we tested this compound in two separate experiments, using the same concentrations in triplicate wells as above. Again, the effect was measured after 24 h and 48 h and the EC_50_ values were determined.

In order to test the influence of cysteine, present in the growth media of both parasites, on the activity of methylgerambullin, media were prepared containing different cysteine concentrations, 0 mg/ml, 0.125 mg/ml, 0.25 mg/ml, 0.5 mg/ml or 1 mg/ml for *E. histolytica* and 0 mg/ml, 0.25 mg/ml 0.5 mg/ml, 1 mg/ml or 2 mg/ml for *G. duodenalis.* The highest values (1 mg/ml for *E. histolytica* and 2 mg/ml for *G. duodenalis* as mentioned above) are the standard cysteine concentrations used in all the previous tests. Then the parasites were cultivated anaerobically in microtiter plates in each of the media with varied cysteine concentrations in the presence of 0 μg/ml (control), 1 μg/ml, 5 μg/ml or 20 μg/ml of methylgerambullin. After 24 h, the remaining cells were counted.

### 2.4 Chemical synthesis of methylgerambullin

**Fig. 2:**
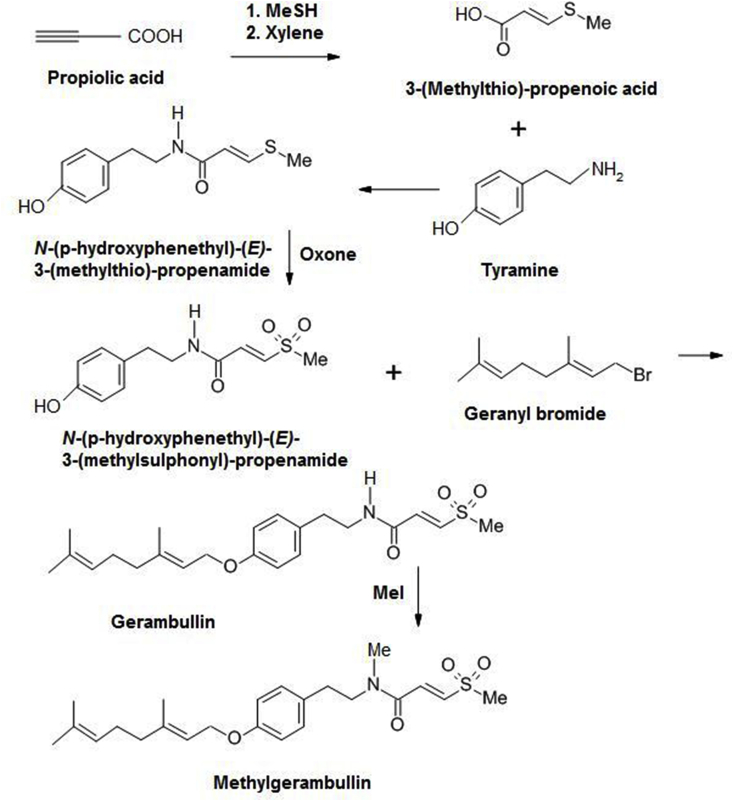
Chemical synthesis of methylgerambullin from commercially available compounds.

Methylgerambullin was synthesised at the company Selvita (Krakow, Poland) in an analogous way to the synthesis of methylgerambullone (Moon et al., 2010). Briefly (Fig. 2), propiolic acid and methanethiol were condensed and isomerised in xylene to form 3-(methylthio)-(*E*)-propenoic acid (yield: 66%). An amide of this compound with tyramine was formed resulting in *N*-(p-hydroxyphenethyl)-(*E*)-3-(methylthio)-propenamide (yield: 52%). This compound was then oxidised to the sulfone (yield 67%). The geranyl group was introduced by geranyl bromide (yield: 69%), and finally the amide nitrogen was methylated using methyliodide (yield: 63%). The purity (>97%) and identity of the synthesised compound were assessed by HPLC and ^1^H and ^13^C NMR (Supplementary Figs. S1 and S2), and the data corresponded to the original results from the plant-derived compound (Greger et al., 1994).

## 3 Results

### 3.1 Selection of compounds

Fourteen purified compounds of various chemical classes and from different plant families were tested (Fig. 1, Table 1). Total plant extracts were not included, because it is easier to synthesise known compounds and in the future to generate improved derivatives.

### 3.2 Anaerobic assay and testing of the compounds in quick tests

For the examination of *E. histolytica* the most suitable system was to culture the amoebae in microtiter plates in an anaerobic environment created by Anaerocult A sachets in air-tight plastic boxes (results not shown), also *G. duodenalis* was examined in this anaerobic environment. All the compounds were tested in quick tests at final concentrations of 2.5 μg/ml and 10 μg/ml, and the cells were counted after 24 h and 48 h. The results are shown in Table 2. A (*E. histolytica*) and 2. B (*G. duodenalis*). Taken together, the compound methylgerambullin stood out as most active against both *E. histolytica* and *G. duodenalis*. At the higher concentration *E. histolytica* was inhibited by 96.5% after 24 and 96.8% after 48 h. *G. duodenalis* at the same concentration was inhibited by 97% after 24 h and by 99.5% after 48 h. In contrast, the other sulphur-containing amides methyldambullin and sakambullin were much less active. Whereas the compound aglafoline displayed significant and the second best activity against *G. duodenalis*, it was ineffective against *E. histolytica*.

**Table 2:** Quick tests of the compounds against *E. histolytica* and *G. duodenalis*.

A - Test of growth inhibition (GI) [%] of *E. histolytica*.				
Assay time	24 h		48 h	
Concentration	10 μg/ml	2.5 μg/ml	10 μg/ml	2.5 μg/ml
Aglafoline	16.3 ± 1.9	11.4 ± 4.2	7.8 ± 15.2	6.7 ± 6.3
Dictamnine	6.2 ± 13.2	−5.4 ± 12.8	15.7 ± 3.3	−6.4 ± 7.2
Iso-gamma-fagarine	5.9 ± 12.8	27.4 ± 21.2	16.1 ± 3.7	−28.7 ± 11.3
Kokusagenine	8.8 ± 2.1	21.4 ± 5.1	0 ± 8.4	−25.8 ± 27.8
Yukocitrine	11.1 ± 15.4	15.8 ± 9.3	−41.9 ± 13.7	−21.7 ± 14.0
Arborinine	15.3 ± 12.1	32.4 ± 5.6	−33.2 ± 4.3	−57.3 ± 19.4
5-Hydroxynoracronycine	31.1 ± 5.9	26.6 ± 2.2	26.4 ± 6.1	−41.8 ± 4.8
Zanthobungeanine	28.7 ± 4.2	9.7 ± 4.7	2.3 ± 17.2	−7.1 ± 7.0
Arborine	22.9 ± 9.8	36.6 ± 7.3	−51.5 ± 9.5	−34.7 ± 14.7
Microminutine	26.5 ± 2.9	14.5 ± 1.6	−6.6 ± 9.7	13.4 ± 9.2
Methyllacarol	−13.6 ± 14.5	2.8 ± 11.1	−16.1 ± 13.5	−18.9 ± 9.8
Methyldambullin	27.4 ± 12.8	13.9 ± 9.8	−1.0 ± 6.0	−24.3 ± 21.6
Sakambullin	25.9 ± 10.4	8.1 ± 13.5	−13.6 ± 25.5	−56.8 ± 9.5
Methylgerambullin	96.5 ± 2.3	48.2 ± 14.9	96.8 ± 0.3	30.9 ± 4.9

B - Test of growth inhibition (GI) [%] of *G.duodenalis*				
Assay time	24 h		48 h	
Concentration	10 μg/ml	2.5 μg/ml	10 μg/ml	2.5 μg/ml
Aglafoline	62.6 ± 5.5	63.7 ± 2.6	73.9 ± 2.5	75.4 ± 2.8
Dictamnine	24.0 ± 9.5	11.8 ± 1.6	1.7 ± 3.5	0.5 ± 4.2
Iso-gamma-fagarine	36.2 ± 5.5	0.1 ± 3.14	2.7 ± 3.3	−1.8 ± 2.2
Kokusagenine	18.4 ± 7.9	24.7 ± 1.4	36.1 ± 3.5	28.9 ± 2.3
Yukocitrine	7.5 ± 4.6	14.9 ± 9.3	7.7 ± 2.8	3.7 ± 10.1
Arborinine	17.0 ± 2.1	5.7 ± 3.8	12.2 ± 5.5	13.2 ± 3.6
5-Hydroxynoracronycine	−2.1 ± 14.1	−0.1 ± 11.3	18.2 ± 5.4	16.1 ± 4.2
Zanthobungeanine	28.2 ± 7.4	0.1 ± 6.7	41.1 ± 7.8	40.1 ± 7.8
Arborine	27.8 ± 3.4	18.6 ± 4.8	9.1 ± 1.6	7.5 ± 3.7
Microminutine	21.5 ± 10.0	10.4 ± 7.7	−2.9 ± 6.5	−5.6 ± 9.6
Methyllacarol	27.9 ± 10.0	15.8 ± 5.3	19.2 ± 4.2	26.8 ± 13.9
Methyldambullin	54.8 ± 4.4	21.2 ± 11.7	26.4 ± 12.3	3.6 ± 5.0
Sakambullin	7.2 ± 2.2	7.1 ± 3.5	10.8 ± 1.9	0.7 ± 3.4
Methylgerambullin	96.9 ± 2.0	54.1 ± 12.9	99.5 ± 0.5	36.9 ± 9.3

The assays were carried out in triplicates in 96-well microplates in an air-tight plastic box under anaerobic conditions. The parasites were seeded at a concentration of 40,000 cells ml^−1^ in a volume of 300 μl. After incubation of 24 h or 48 h a sample was stained with Trypan blue and the number of the dead and living cells was counted. GI [%] (percent growth inhibition) ± standard deviation *σ*_n_ [%] was calculated as described in Materials and Methods.

### *3.3* Activity of methylgerambullin against *E. histolytica* and *G. duodenalis* and aglafoline against *G. duodenalis*

The sulphur-containing amide methylgerambullin, which had shown good activity against both protozoans, was tested at several concentrations and the cells were counted in three experiments each either after 24 h or after 48 h. The experiments were carried out with metronidazole as a control. The EC_50_ results are shown in Table 3. A (*E. histolytica*) and Table 3. B (*G. duodenalis*). The EC_50_s for *E. histolytica* after 24 h and 48 h were 14.5 μM (6.08 μg/ml) and 17.4 μM (7.33 μg/ml), respectively. The EC_50_s for *G. duodenalis* after 24 h and 48 h were 14.6 μM (6.14 μg/ml) and 36.5 μM (15.34 μg/ml), respectively. For unknown reasons, the first experiment on *G. duodenalis* showed rather poor activity (not shown) which increased the calculated mean. Taken together, the activity of methylgerambullin against both parasites was comparable.

**Table 3:** Activity of methylgerambullin against *E. histolytica* (A) and methylgerambullin and aglafoline against *G. duodenalis* (B).

A - Entamoeba histolytica	G [μM]	*σ*_g_
Methylgerambullin		
24 h	14.5	1.36
48 h	17.5	1.59
Control metronidazole		
24 h	2.40	
48 h	1.40	
B - Giardia duodenalis	G [μM]	*σ*_g_
Methylgerambullin		
24 h	14.6	2.85
48 h	36.6	6.08
Aglafoline		
24 h	17.2	
48 h	7.71	
Control metronidazole		
24 h	3.15	
48 h	1.93	

Test of methylgerambullin and metronidazole (control) against *E. histolytica* (A) as well as methylgerambullin, aglafoline and metronidazole (control) against *G. duodenalis* (B). The geometric means *G* [μM] of EC_50_ values are shown for three experiments (methylgerambullin) or two experiments (metronidazole, aglafoline), in addition the geometric standard deviations *σ*_*g*_ are given where three experiments were performed.

The calculated EC_50_s for the metronidazole after 24 h and 48 h were 2.40 μM (0.41 μg/ml) and 1.40 μM (0.24 μg/ml) for *E. histolytica* (Table 3. A) and 3.16 μM (0.54 μg/ml) and 1.93 μM (0.33 μg/ml) for *G. duodenalis* (Table 3. B).

Although only methylgerambullin was active against both parasites, aglafoline did exhibit a significant activity against *G. duodenalis* in the quick tests (Table 2. B). With the remaining amount of aglafoline we were able to perform two EC_50_ measurements with triplicate samples, and the outcome is presented in Table 3. B, the EC_50_ for 24 h was 17.2 μM (8.47 μg/ml), and for 48 h as low as 7.71 μM (3.80 μg/ml).

### 3.4 Influence of the concentration of cysteine on the activity of methylgerambullin

An ample supply of sulphur is provided as cysteine in media for *E. histolytica* (1 mg/ml) as well as *G. duodenalis* (2 mg/ml). We hypothesised that cysteine could influence the activity of methylgerambullin as it does for the activity of metronidazole (Leitsch et al., 2007). Therefore the activity of the sulphur-containing compound methylgerambullin in concentrations of 0 μg/ml, 1 μg/ml, 5 μg/ml or 20 μg/ml, respectively, was tested against both protozoans cultivated in media containing different cysteine concentrations, 0 mg/ml, 0.125 mg/ml, 0.25 mg/ml, 0.5 mg/ml or 1 mg/ml for *E. histolytica* and 0 mg/ml, 0.25 mg/ml 0.5 mg/ml, 1 mg/ml or 2 mg/ml for *G. duodenalis.* The surviving parasites were counted after 24 h.

**Fig. 3:**
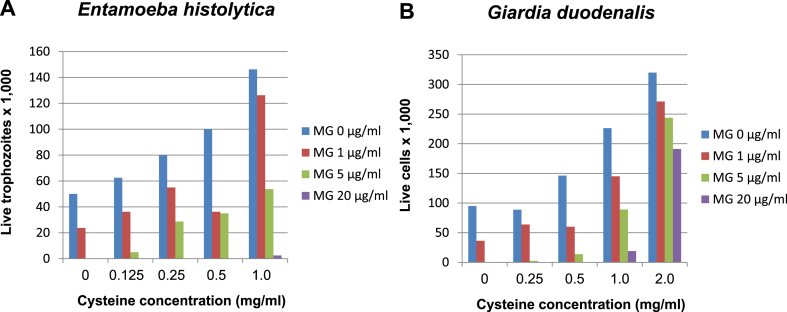
**A.***Entamoeba histolytica* was cultivated for 24 h with different concentrations of methylgerambullin (MG) in medium with different cysteine concentrations and the number of surviving trophozoites was counted. **B.** The same experiment was performed for *G. duodenalis*.

The results are shown in Fig. 3A and 3B. Clearly, the activity of methylgerambullin (MG) is inhibited in rich media with high cysteine concentrations. Of course the growth of *E. histolytica* and *G. duodenalis* is also lower as less cysteine is present in the medium. When we look at the cysteine concentrations of 0.25 mg/ml for *Entamoeba* and 0.5 mg/ml for *Giardia* we can see, however, that the untreated cells still have enough cysteine to grow and divide successfully and at the same time even lower concentrations of methylgerambullin show good efficacy.

### 3.5 Chemical synthesis of methylgerambullin

As among the sulphur-containing amides methylgerambullin stood out as the most active, it was desirable to be able to synthesise the compound and in the future to be able to generate derivatives as well. Fortunately, the compound could be synthesised rather easily from the commercially available compounds propriolic acid, methanethiol, tyramine and geranyl bromide (section 2.4 and Fig. 2).

## 4 Discussion

### 4.1 The sulphur-containing amide methylgerambullin and its origin

The fourteen compounds which were tested in this study against *E. histolytica* and *G. duodenalis* belong to seven different chemical classes and are shown in Fig. 1 and listed in Table 1. In preliminary tests with 2.5 μg/ml and 10 μg/ml (Tables 2A and 2B), the sulphur-containing amide methylgerambullin had the highest activity against both parasites. Therefore, the rest of this study was mainly focussed on this compound, although aglafoline had some lesser activity against *G. duodenalis*, but not against *E. histolytica*. The EC_50_s of methylgerambullin against *E. histolytica* (Table 3. A) after 24 h and 48 h were 14.5 μM (6.08 μg/ml) and 17.4 μM (7.33 μg/ml), respectively and the EC_50_s against *G. duodenalis* (Table 3. B) after 24 h and 48 h were 14.6 μM (6.14 μg/ml) and 36.5 μM (15.34 μg/ml), respectively. The EC_50_ of aglafoline against *G. duodenalis* after 24 h and 48 h was 17.2 μM (8.47 μg/ml) and 7.71 μM (3.80 μg/ml), respectively.

Methylgerambullin and the other sulphur-containing amides methyldambullin and sakambullin are found in the leaves of *Glycosmis* spp., a small genus of about 40 species in the family Rutaceae (*Citrus* plants). The plants grow in shrubs or small trees and develop small berries typically with a sweet taste giving the genus its name. In contrast, the leaves, from which the sulphur-containing amides were extracted, appear to have an unpleasant taste for grazing animals.

Chemically, the sulphur-containing acid moiety 3-(methylsulfonyl)-propenoic acid could be derived from cysteine and the p-hydroxyphenethyl amide part could be derived from tyrosine. This structure is linked to prenyloxy side chains in methyldambullin and sakambullin and to a geranyloxy side chain in methylgerambullin (Fig. 1) (Hofer and Greger, 2000). The sulphur in these prenylated amides is mostly oxidized to a sulfone or sulfoxide (Hofer et al., 2000).

### 4.2 Bioactivities of the sulphur-containing amides

The sulphur-containing amides methylgerambullin, sakambullin and methyldambullin were all highly active against *T. cruzi* epimastigotes (Astelbauer et al., 2010). Methylgerambullin had the lowest EC_50_ of 2.83 μM after 48 h of treatment, compared to 4.50 μM and 4.17 μM for sakambullin and methyldambullin. In a second study on *L. infantum* promastigotes, methyldambullin had an EC_50_ of 1.1 μM after 48 h of treatment (Astelbauer et al., 2011), and later, the activity of methylgerambullin after 48 h was tested at an EC_50_ of 0.56 μM (Astelbauer, unpublished data).

In further preliminary studies, methylgerambullin strongly inhibited the maturation of *Plasmodium falciparum* schizonts, but was inactive against *Trichomonas vaginalis* (Astelbauer, unpublished data).

Methylgerambullin showed cytotoxic activity against CEM-SS (T-lymphoblastic leukaemia), KU812F (chronic myelogenous leukaemia), HT29 (colon cancer) and UACC-62 (melanoma) cell lines, however, methylgerambullin was much less toxic against human peripheral blood mononuclear cells (Mohamed et al., 2000). Also, methylgerambullin showed no acivity against fish-pathogenic bacteria (Abdullah et al., 2006).

Taken together, methylgerambullin had a broad activity against protozoan parasites, and was superior to the other two sulphur-containing amides.

### 4.3 Properties of methylgerambullin

A potential orally active drug should be soluble and able to permeate to reach its target. Lipinski's “Rule of 5” (Lipinski et al., 1997) has become a widely-used tool to assess these desired properties. The rule states, that drug-like molecules should have a logP ≤5 (a measure of hydrophobicity), a molecular mass ≤ 500 Da, the number of hydrogen bond acceptors should be ≤ 10 and the number of hydrogen bond donors ≤ 5. We used the web tool www.molinspiration.com provided by the company Molinspiration Cheminformatics (Slovensky Grob, Slovak Republic) to test if the three sulphur-containing amides conform to the Rule of 5, and they all do. Although conforming to the Rule of 5 is a positive property of a molecule, many valuable drugs, in particular natural products, but sometimes even compounds designed by medicinal chemistry, do not conform to the Rule of 5 (Lipinski, 2016).

**Fig. 4:**
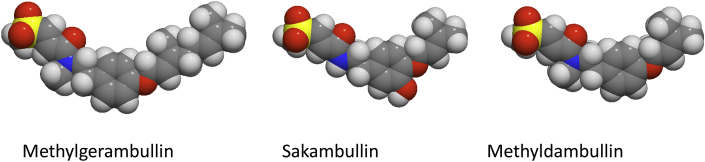
Predicted structures of the sulphur-containing amides as generated by the Molinspiration Galaxy 3D Structure Generator (www.molinspiration.com).

The Molinspiration website also provides the Galaxy 3D Structure Generator, which allows to visualize molecules without very unusual properties. The predicted structures of the three sulphur-containing amides are shown in Fig. 4. All three structures are bent, and there is a more hydrophilic part containing the sulfone and a more hydrophobic part with the prenyl or geranyl portion. In contrast to methylgerambullin, the compound methyldambullin with the same sulphur-containing amide part had much less activity against *G. duodenalis* and no activity against *E. histolytica*. Possibly the longer hydrophobic geranyl portion of methylgerambullin could interact with the plasma membrane of the parasites more strongly resulting in a higher activity.

A large advantage of the compounds is that they do not contain chiral centres and they can be synthesised from few commercially available starting materials. So it will be rather easy to generate derivatives from the compounds, such as increasing the hydrophobic side chain length to a farnesyl group. This might on the one hand increase the activity, but the logP calculation shows that the increased hydrophobicity does no longer conform to the Rule of 5.

Hydrophobicity of methylgerambullin was observed in this study. The compound dissolves extremely well in dimethylsulfoxide (DMSO) (>600 mg/ml), but no useful concentrations can be generated in H_2_O or ethanol. In contrast, it was possible to dissolve methylgerambullin at a concentration of 10 mg/ml in the non-toxic liquid polymer polyethylene glycol 300 (PEG 300). This required stirring overnight, however. Taken together, there will be few obstacles to synthesise a range of derivatives for methylgerambullin, but significant efforts will be needed to find the most suitable ones.

### 4.4 Influence of cysteine on the activity of methylgerambullin - a possible link to its mode of action

**Fig. 5:**
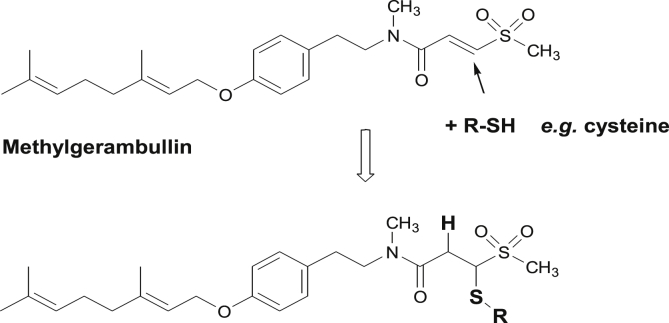
Proposed reaction scheme for the reaction of methylgerambullin with cysteine.

Cysteine, the important component of *E. histolytica* and *G. duodenalis* media, serves as an anti-oxidant but at least as importantly, as a source of sulphur for the biosynthesis of cysteine-rich proteins as well as iron-sulphur clusters. Under anaerobic conditions, however, *E. histolytica* proliferates better with cystine than with cysteine, and can do altogether without cysteine. In contrast, *G. duodenalis* needs cysteine, but the high concentrations are required due to the concomitant presence of bile in the medium, and without bile, *G. duodenalis* could do with much less cysteine (Leitsch, 2017). Importantly, cysteine inhibits the activity of various anti-parasitic compounds (Leitsch, 2017). So this was also the case for methylgerambullin (Fig. 3A and Fig. 3B). Whereas 5 μg/ml of methylgerambullin were sufficiently active at low cysteine concentrations, the standard medium concentrations of 1 mg/ml for *E. histolytica* and even more the 2 mg/ml for *G. duodenalis* drastically inhibited the drug activity. It may be imagined that cysteine is able to react with methylgerambullin (Fig. 5). In addition, this type of reaction could also occur with other free thiols in the parasite. The consequences could be inactivation of the drug or, alternatively, inactivation of proteins possessing reduced cysteine residues.

Inside the colon, *E. histolytica* has to compete for cysteine with various bacteria with an active metabolism of sulphur compounds, such as bacteria harbouring cysteine desulphydrase, which degrades cysteine to pyruvate, ammonia and hydrogen sulphide (Carbonero et al., 2012). When the amoebae invade the colonic mucosa, they encounter a cysteine concentration decreasing along the colon from about 27 mg/kg tissue in the ascending colon to about 13–15 mg/kg tissue in the transverse and descending colon and rectum (Ahlman et al., 1993). Amoebae invading into the bloodstream will encounter a plasma cysteine concentration of about 27 μg/ml (Ahlman et al., 1993). Taken together, these cysteine concentrations are much too low to significantly inhibit the activity of methylgerambullin.

### 4.5 On the activity of aglafoline against *G. duodenalis*

Although aglafoline was active only against *G. duodenalis* in this study, it should still be considered. It obeys Lipinski's “Rule of 5”, but it will be much more difficult to sythesize than methylgerambullin, as aglafoline possesses five centres of chirality. In our older studies it had shown activity against *L. infantum* (Astelbauer et al., 2011) and *P. falciparum* (Astelbauer et al., 2012), but not against *T. cruzi*.

### 4.6 Conclusions

From a panel of 14 plant compounds, methylgerambullin was identified as the compound with the highest activity against *E. histolytica* and *G. intestinalis*. The activity was lower than that of metronidazole, but part of the reason was that cysteine in the culture media of both parasites inhibited the action. The chemical synthesis of the compound is straightforward and this puts the discovery of derivatives with higher activity and favourable pharmacological properties within reach. The compound aglafoline was active only against *G. duodenalis* but should also be considered in the future.

## Declarations of interest

None.
