# Supplementary material for: Activity of methylgerambullin from Glycosmis species (Rutaceae) against Entamoeba histolytica and Giardia duodenalis in vitro
Source: Int J Parasitol Drugs Drug Resist. 2019 Aug 10;10:109–17. doi: 10.1016/j.ijpddr.2019.08.001 (PMC6722286; doi:10.1016/j.ijpddr.2019.08.001)
Supplement: Multimedia component 2 [file mmc2.docx]

**Supplementary Figures**


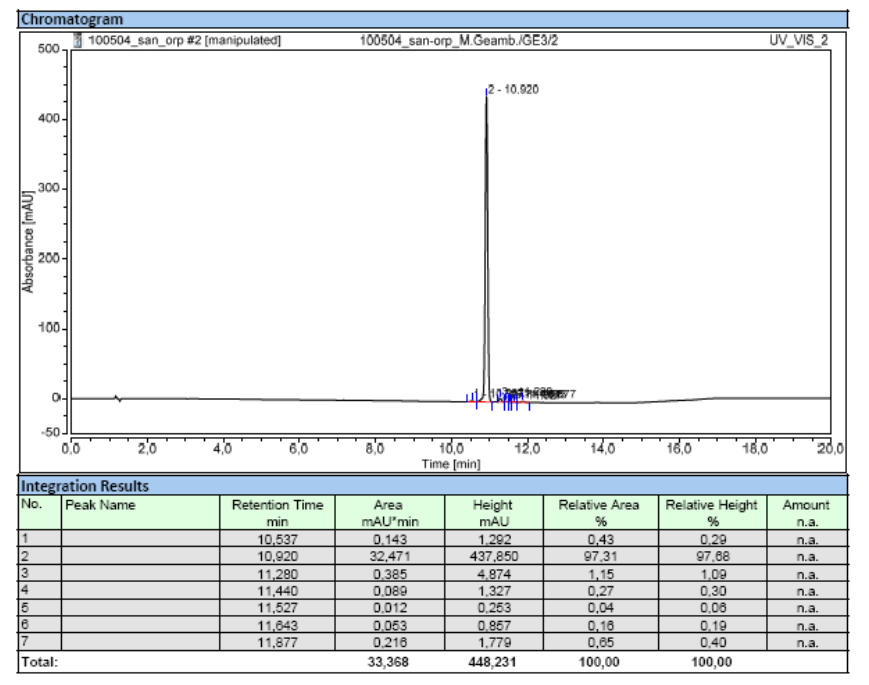


**Supplementary Figure S1:** HPLC of chemically synthesized methylgerambullin. A 3 ml sample was injected and run for 20 min. The absorbance was monitored at a wavelength of 250 nm.


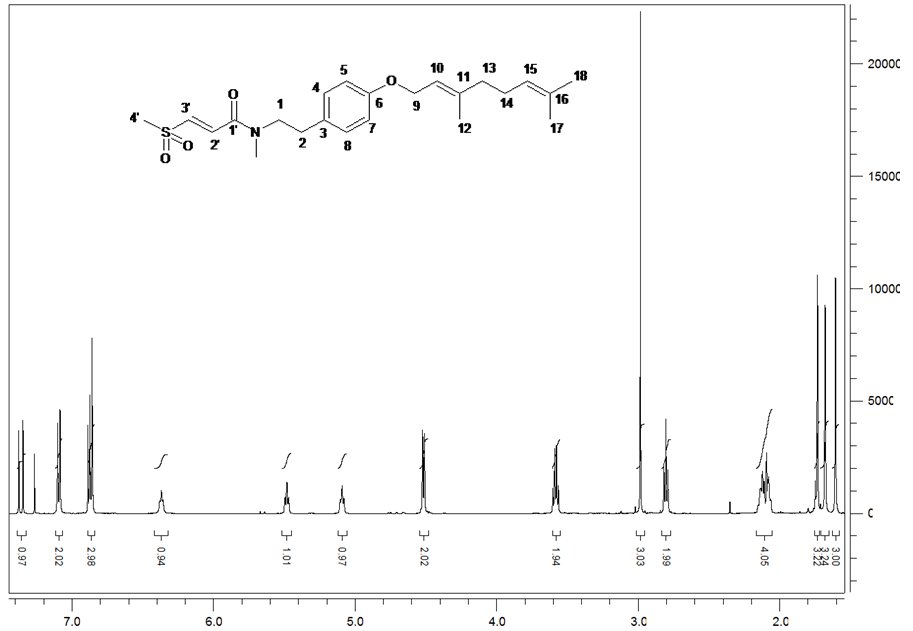


ppm


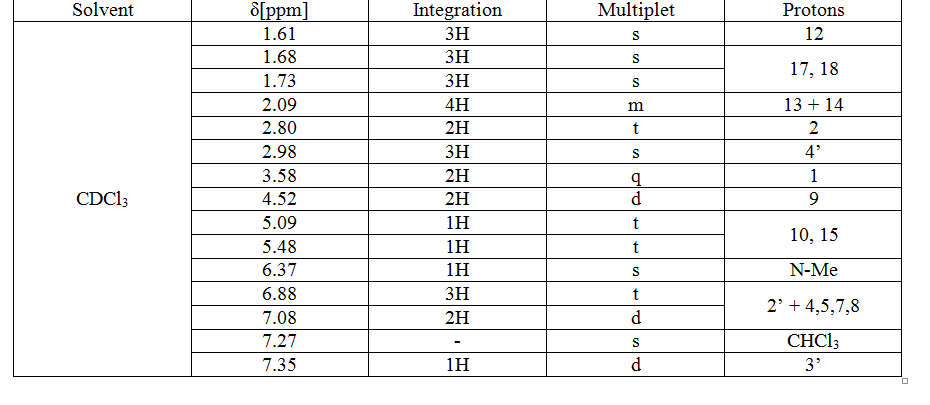


**Supplementary Figure S2:** ^1^H NMR of chemically synthesized methylgerambullin in CDCl_3_. The annotation is given in the table of shifts. The spectrum corresponds to the ^1^H NMR of plant-derived methylgerambullin (Greger et al., 1994).
